# A randomized, open-label, multicentre, comparative study of therapeutic efficacy, safety, and tolerability of BNO 1030 extract, containing marshmallow root, chamomile flowers, horsetail herb, walnut leaves, yarrow herb, oak bark, dandelion herb, in the treatment of mild forms of COVID-19

**DOI:** 10.1186/s40816-021-00308-x

**Published:** 2021-08-28

**Authors:** Vasyl Popovych, Ivana Koshel, Yulia Haman, Vitaly Leschak, Oleksandr Malofiichuk, Natalia Kapustina, Ihor Shevaga, Olha Shevaga, Tetiana Kunytska

**Affiliations:** 1 Department of ENT Diseases, Head and Neck Surgery, Ivano-Frankivsk National Medical University, Ivano-Frankivsk, Ukraine; 2 Department of Therapy and Family Medicine, Institute of Postgraduate Education, National Medical University, Ivano-Frankivsk, Ukraine; 3 Uzhgorod National University, Uzhhorod, Ukraine; 4 Regional Clinical Hospital of Gerbachevsky, Ivano-Frankivsk, Ukraine; 5 Kamianets-Podilsky City Hospital , Kamianets-Podilsky, Ukraine

**Keywords:** Phytotherapy, BNO 1030, Imupret®, Mild COVID-19

## Abstract

**Background:**

COVID19 is a high burden for medicine and society as still no specific therapy exists. Most patients depend on symptomatic treatment, comparable to the symptomatic treatment in common respiratory infection e.g., Acetaminophen or Ibuprofen. Many cases of COVID19 show mild forms without need of hospitalization. In this randomized, open-label, multicentre, comparative trial we analysed the efficacy, safety, and tolerability of the herbal medicinal product BNO 1030 in mild cases of COVID-19 to offer an additional symptomatic relive.

**Methods:**

The study was designed as an open label randomized, prospective, multicentred clinical trial. Out of 133 screened outpatients aged 18 to 70 with mild COVID-19 symptoms 120 patients were randomised (1:1) in 2 parallel groups. The main group received BNO 1030 in addition to symptomatic therapy (acetaminophen or ibuprofen). The control group got a symptomatic therapy only. The patients with laboratory proven COVID 19 were included for the final analyses: 47 – in the main group and 46 – in the control one. The evaluation criteria were dynamics of the symptoms: hyperthermia, myalgia, nasal congestion, nasal discharge, coughing, anosmia, rhinolalia, sore throat, duration of the use of antipyretics (clinically significant fever). These symptoms were assessed during the physician’s visit on a 4-point scale (0 — absent, 1 — insignificant, 2 — moderate, 3 — strong/pronounced) and self- assessed via ten-point visual analogue scale (VAS) daily in a patient’s diary. The primary endpoint was the decrease of the average score compared to the baseline defined as “therapeutic benefit” from the usage of BNO 1030.

**Results:**

In the comparison of both groups over the treatment time, the main group (*n* = 47) showed a greater decrease in the severity of symptoms of fever, myalgia, nasal congestion, coughing, anosmia and rhinolalia, assessed by the doctor on a 4-point scale on V2 (4th day) and V3 (14th day) compared to those on V1, as well as a reduction of the antipyretics intake duration (*p* < 0.05). Significant differences of the main group were obtained, too, based on the results of symptoms self-assessment by the patient. The “therapeutic benefit from the use of BNO 1030 was 3 days. There is an increase in the number of recovered patients from 73.9–96.6 % according to the average symptom score, and a decrease in the number of hospitalized patients from 8.6–4.4 % in the main group., as compared to the data of the control group (*p* < 0.05). All patients tolerated the herbal medicine well, with no adverse drug reactions being reported.

**Conclusions:**

BNO 1030 (Imupret®) offers a safe and effective treatment benefit in patients with mild forms of diagnosed COVID-19 aged 18–70 in addition to symptomatic treatment with acetaminophen or NSAIDs. COVID 19 positive patients treated with Imupret showed an earlier relive of symptoms when being treated with BNO 1030.

**Trial registration:**

This trial was registered in ClinicalTrial.gov: NCT04797936.

## Introduction

For patients with mild COVID-19, who are not included in the risk factor groups, WHO and national guidelines recommend outpatient treatment in compliance with the self-isolation regimen. Treatment recommendations include diet, the usage of antipyretics (paracetamol, ibuprofen) for hyperthermia and pain [1, 2], and other medications for symptomatic therapy (if indicated).

However, these symptomatic agents do not cover the entire spectrum of pathogenetic mechanisms of inflammation associated with COVID-19. Furthermore, several trials have expressed concern that ibuprofen might lead to a more severe course of coronavirus disease, although there is no convincing evidence that could either confirm or refute this [3, 4]. Given this, it is necessary to use combination drugs. From this perspective, certain plant compounds could be an important source of medicinal products for the treatment of COVID-19 during the pandemic. Repurposing of medicinal products has several potential benefits, including reduced development times, lower costs, and regulatory support for faster market launch of treatment which could alleviate the current pandemic. An experimental *in vitro and in silico* study has shown that the combination of curcumin with pipeline has antiviral activity against SARS-CoV-2 [5]. Herbal medicinal products have the potential to interfere with various steps of the viral replication cycle and/or may be able to strengthen healing and regeneration processes by modulating the host’s immune response in a multimodal manner. Furthermore, herbal medicinal products are well-tolerated due to their low rate of adverse reactions. Many herbal components, e.g. flavonoids, terpenoids, polysaccharides or diverse glycosylated metabolites demonstrate potency against respiratory and inflammatory diseases due to either direct anti-viral or anti-inflammatory effects. In herbal extracts, consisting of a multitude of molecular components, various anti-viral actions may be combined to act in an additive or even synergistic manner. In September 2020, the WHO expert committee approved a protocol for clinical trials of medicinal herbs for the treatment of COVID-19 [6]. Herbal medicinal products have the potential to interfere with various steps of the viral replication cycle and/or may be able to strengthen healing and regeneration processes by modulating the host’s immune response in a multimodal manner. Furthermore, herbal medicinal products are well-tolerated due to their low rate of adverse reactions. Many herbal components, e.g. flavonoids, terpenoids, polysaccharides or diverse glycosylated metabolites demonstrate potency against respiratory and inflammatory diseases due to either direct anti-viral or anti-inflammatory effects. In herbal extracts, consisting of a multitude of molecular components, various anti-viral actions may be combined to act in an additive or even synergistic manner Obviously, there are very few clinical studies on the effectiveness of herbal medicines which meet the GCP standards, but nevertheless, the situation has changed after the release of the relevant recommendations [7]. As less attention is paid to mild course cases, although current data show that 40–80 % of patients diagnosed with COVID-19 feature a mild course of the disease [8, 9], this trial elucidates the potential of herbal medicinal products with a proven safety as an optimization of the symptomatic treatment for mild COVID 19 cases. Considering the recent emergence of mutated virus strains, the reproductive rate of SARS-COV2 number is increasing and vaccination procedures are slow. The proportion of asymptomatic disease forms among people infected with COVID-19 is high, and the transmission potential is significant [10, 11]. Moreover, possible progression of a COVID-19 mild form remains an urgent issue since variants of a severe course of the disease originate from relatively mild symptoms. A shorter period from the onset of symptoms to their worsening and hospitalization is associated with a worse outcome in patients with COVID-19, and we still have insufficient information on the actual course and treatment of mild forms of the disease [12, 13]. At the same time, WHO, in its updated tactics to contain the COVID-19 spread, declares the need for diagnosis and effective treatment of patients with mild to moderate severity of the disease [1]. The mild course of COVID-19 is characterized by some non-specific, symptoms comparable to a common cold or in influenza infection, i.e. fever, coughing, sore throat, nasal congestion, sickliness, headache, myalgia, anosmia in the absence of symptoms of pneumonia and oxygen deficiency [1, 14]. These symptoms are typical for non-severe acute viral infections of the upper respiratory tract, acute nasopharyngitis, caused by already known human coronaviruses such as hCoV-229E, OC43, NL63 and HKU1 [15, 16]. Unfortunately, there is no effective treatment for COVID-19. The mechanisms of potential medicinal products are associated with antiviral action (remdesivir, lopinavir, ritonavir, interferon beta), blockade of the virus fusion with the cell membrane — recombinant human angiotensin-converting enzyme, hydroxychloroquine, and some others, but they are considered for treatment of severe forms of the disease [17–19]. Some of these tested drugs did not show a clinical benefit [20–22].

In September 2020, the WHO expert committee approved a protocol for clinical trials of medicinal herbs for the treatment of COVID-19 [6]. Herbal medicinal products have the potential to interfere with various steps of the viral replication cycle and/or may be able to strengthen healing and regeneration processes by modulating the host’s immune response in a multimodal manner. Furthermore, herbal medicinal products are well-tolerated due to their low rate of adverse reactions. Many herbal components, e.g. flavonoids, terpenoids, polysaccharides or diverse glycosylated metabolites demonstrate potency against respiratory and inflammatory diseases due to either direct anti-viral or anti-inflammatory effects. In herbal extracts, consisting of a multitude of molecular components, various anti-viral actions may be combined to act in an additive or even synergistic manner Obviously, there are very few clinical studies on the effectiveness of herbal medicines which meet the GCP standards, but nevertheless, the situation has changed after the release of the relevant recommendations [7]. Clinical practice uses the phytoneering extract BNO 1030 consisting of seven medicinal plants, namely: Marshmallow root (Radix Althaeae), Cammomile flowers (Flores Chamomillae), Horstail herb (Herba Equiseti), Walnut leaves (Folia Jungladis), Yarrow herb (Herba Millefolii), Oak bark (Cortex Quercus), Dandelion herb (Herba Taraxaci), known as Imupret® (known in some countries as Tonsilgon® N). The components of the medicinal product provide an antiviral, antibacterial, anti-inflammatory effect and promote the activation of non-specific immunity factors [23–29]. The indications for use are “the treatment of the upper respiratory tract diseases and prevention of complications and relapses in viral respiratory infections”. Currently, experience has been accumulated on the use of Imupret for the treatment of acute viral infections of the pharyngeal lymphoid ring: acute nasopharyngitis and tonsillitis [30, 31]. This study aimed at evaluating the efficacy of the BNO 1030 herbal extract (Imupret®) as an additional therapy to standard treatment compared to that of the standard symptomatic therapy alone for mild COVID-19 (acute nasopharyngitis) according to the WHO and national guidelines [1, 2].

## Materials and methods

### Trial design

The open-label, exploratory, comparative, multicentre, randomized, prospective, parallel-group study took place in four outpatient departments affiliated with hospitals in Ukraine from June 2020 to December 2020. The study was conducted following the GCP standards and the Declaration of Helsinki and approved by the Ethics Committee at all research centres. Each study participant provided written consent to participate in the study before performing any research-related procedures.

### Participants

The study provided for screening 133 patients with symptoms of mild form of COVID-19 infection and randomizing 120 outpatients aged 18–70 who were diagnosed with mild COVID-19 based on clinical data: clinical symptoms and previous contact with a person with confirmed n-Cov-19 infection. Randomly all the patients were divided into two groups: the main group taking BNO 1030, a standardized extract of seven medicinal plants (Imupret®) in addition to the standard therapy, and the control group receiving the standard symptomatic therapy alone. 18 men (30.0 %) and 42 women (70.0 %) (mean age 32.98 ± 13.12) were randomized into the main group (*n* = 60), and 19 men (31.7 %) and 41 women (68.3 %) (mean age 33.90 ± 11.79) — into the control group (*n* = 60).

Inclusion criteria: males and females aged 18 to 70, with typical disease symptoms of COIV19. The must have symptoms were sudden onset of the symptoms, olfactory disorder (anosmia, hyposmia) and documented contact with a confirmed COVID19 case. Further typical disease symptoms which can be hyperthermia, myalgia, coughing, nasal congestion, nasal discharge (anterior or posterior rhinorrhoea) depending on the prevalence of the disease. Further inclusion criteria for this study were no signs of viral pneumonia or hypoxia,, the possibility of outpatient self-isolation, the patient’s willingness, and ability to comply with the research protocol, signed informed consent. Clinical symptoms in the inclusion criteria met the diagnostic criteria for mild COVID-19 and complied with the WHO recommendations presented in national clinical guidelines [1, 20]. The randomized patients underwent laboratory verification of COVID-19. The diagnosis was considered confirmed if there was at least one positive PCR, IgM, and IgG result.

Non-inclusion criteria: indications for inpatient treatment, presence of immunodeficiency states, oncological diseases, chronic diseases of the cardiovascular or bronchopulmonary systems, diabetes mellitus, individual intolerance to the medicinal product components.

Criteria for excluding a patient from the study: the patient’s decision to discontinue participation in the study, and withdrawal of the written informed consent, loss of contact with the patient, individual intolerance to the medicinal product under study and the reference treatment regimen, occurrence of serious and/or unforeseen side effects / reactions in the patient during the study, development of complications an underlying medical condition which, in the physician’s opinion, requires the patient to be excluded from the trial, negative laboratory test result for coronavirus infection,.

### Interventions

Patients of the two groups in self-isolation were prescribed a sparing diet, elimination of nasopharyngeal irritants, isotonic saline solutions in the nose 4 times a day for 14 days, antipyretics (acetaminophen or ibuprofen according to the national guidelines of the Ukrainian Ministry of Health) (in the presence of clinically significant fever (more than 38^0^С) and/or myalgia > 3 points according to ten-point visual analogue scale (VAS). Patients of the main group were additionally prescribed BNO 1030 (Imupret®) drops for oral administration, from one batch, in the following dosages: 25 drops 6 times a day.

BNO 1030 drops for oral administration are a standardized aqueous-alcoholic extract. *Active substances*: 100 g drops contain 29 g of an alcoholic aqueous extract (extracting agent: ethanol 59 % (V/V) made from the following medicinal plants:

- Marshmallow root (Radix Althaeae) 0.4 g;
- Cammomile flowers (Flores Chamomillae) 0.3 g;
- Horstail herb (Herba Equiseti) 0.5 g;
- Walnut leaves (Folia Jungladis) 0.4 g;
- Yarrow herb (Herba Millefolii) 0.4 g;
- Oak bark (Cortex Quercus) 0.2 g;
- Dandelion herb (Herba Taraxaci) 0.4 g;

*Excipients*: Ethanol 19 % (V/V), purified water.

Name and address of the manufacturer: Bionorica SE, Kerschensteinerstrasse, 11–15, 92,318, Neumarkt, Germany.

The medicinal product is registered in Ukraine and available over the counter. Therefore, the composition, manufacture, packaging and labelling of the medicinal product comply with the principles of good manufacturing practice and the current national requirements of Ukraine. A detailed description covering all aspects concerning the quality and safety of the BNO 1030 drops is part of the relevant product characteristics.

In Ukraine, the approved indications for its use are treatment of upper respiratory tract diseases (tonsillitis, pharyngitis, laryngitis) and prevention of complications and relapses in respiratory viral infections.

Prohibited therapy: other herbal medicines, immune stimulating pharmacological substances, antiviral agents, corticosteroids, other immune stimulating pharmacological substances.

Practising ENT-specialists with at least 5 years of experience took part in the study.

### Outcome measures

All data were assessed by a physician during three visits over 14 days (Table 1).

**Table 1:** Schedule of assessments

V1			V2										V3
day 1	day 2	day 3	day 4	day 5	day 6	day 7	day 8	day 9	day 10	day 11	day 12	day 13	day 14
*Treatment group*													
Reference treatment + Imupret													
*Control group*													
Reference treatment													

V1 day 1 Screening, randomization, prescription of treatment

V2 day 4 ±1 phone contact /or virtual contact— clarification of the patient’s condition, need for an unscheduled visit

V3 day 14 ±1 Evaluation of treatment efficacy, end of treatment

Patient may call on any day. An unscheduled visit can be carried out when the patient’s condition worsens, with the symptoms of the disease persisting or intensifying, including but not limited to an increase in temperature in the armpit above 38.0 °C on the 3rd day and/or subsequent treatment days.

The observation duration for 1 patient was no longer than 14 days ±1 day (the patient’s self-isolation period).

Symptoms, which were included in the manifestation scale of the mild form of COVID-19, such as hyperthermia, myalgia, nasal congestion, nasal discharge, coughing, anosmia, nasal voice, sore throat were evaluated. All the symptoms during the scheduled physician’s visit were assessed on a 4-point scale (0 — absent, 1 — insignificant, 2 — moderate, 3 — strong/pronounced). Hyperthermia was assessed as follows: 0 (absence) < 37 ^0^С, 1 — 37 ^0^С to 37.5 ^0^С, 2 — 37.5 ^0^С to 38 ^0^С, 3 — >38 ^0^С. Moreover, the patient rated his complaints according to the severity of fever, myalgia, nasal congestion, nasal discharge, coughing, anosmia, and sore throat on a ten-point visual analogue scale (VAS) daily in a patient’s diary. The severity of symptoms according to the VAS was assessed as follows: < 3 — mild, 3 to 7 — moderate, > 7 — severe.

The main efficacy criterion was the day of the onset of the response to the treatment, the decrease in the severity of the disease symptoms assessed on a score scale during each visit compared with those during the 1st visit, the dynamics of the physician’s assessment and the patient’s self-assessment of the symptoms. Secondary criteria: dynamics of the use of non-steroidal anti-inflammatory drugs, assessment of the “therapeutic gain” as a result of BNO 1030 use, treatment outcomes, presence or absence of indications for hospitalization.

### Sample size

The study was designed to provide a reliable clinical description of the effectiveness of active (additional) use of BNO 1030 compared to the reference standard treatment alone. Pursuant to the data obtained, several tentative descriptive and statistical evaluations were performed. Based on simplified assumptions, e.g. all patient data are evaluable, alpha = 0.05, applicable two-sided t-test, and equal variances in both groups, the sample size chosen (*N* = 120) is able to detect even weak effect sizes between the treatment groups of 0.37 or more. Treatment allocation was 1:1.

### Randomization

Subjects are randomly assigned to one of the two possible treatments according to the basic randomization list. Randomization was performed using the [StatSoft – random number generator] software. Randomization was performed for each patient who signed an informed consent.

### Statistical methods

To analyze homogeneity of groups, descriptive statistics methods were used for description of the baseline condition of the treatment and control group (for quantitative parameters – n, mean arithmetic, median, standard deviation, minimum and maximum values; for qualitative parameters – incidence and share as %). Verification of normality of data distribution in groups was performed for quantitative parameters using Shapiro-Wilk test. If the data in groups showed normal distribution according to certain parameters, the groups were compared by these parameters via Student’s test for independent samples. Otherwise (if the data distribution was different from normal), comparison of groups was performed according to Mann-Whitney test. For categorical parameters, the groups were compared using Pearson’s chi-squared test or Fisher’s exact test.

For analysis of efficacy, descriptive statistics parameters were calculated in each group (n, mean arithmetic, median, standard deviation, minimum and maximum values) for all visits in accordance with patients’ examination scheme.

Analysis of dynamics of the mentioned parameters in each group was performed via two-way analysis of variance (ANOVA) according to the following scheme: “Visit” factor is fixed (levels: visit 1… visit n); “Subjects” factor is random.

Results of the subsequent visits were compared against the data of visit 1 via contrast analysis using simple contrasts.

Comparison between groups in dynamics of tested parameters was performed by differences dTi = (ТVisit n – ТVisit 1) of assessed parameters using Mann-Whitney test.

The level of confidence for Shapiro-Wilk test was accepted equal to 0.01, and for the rest of the criteria it was accepted equal to 0.05.

The analysis was performed in software environment IBM SPSS 22.0.

## Results

### Study sample

**Fig. 1:**
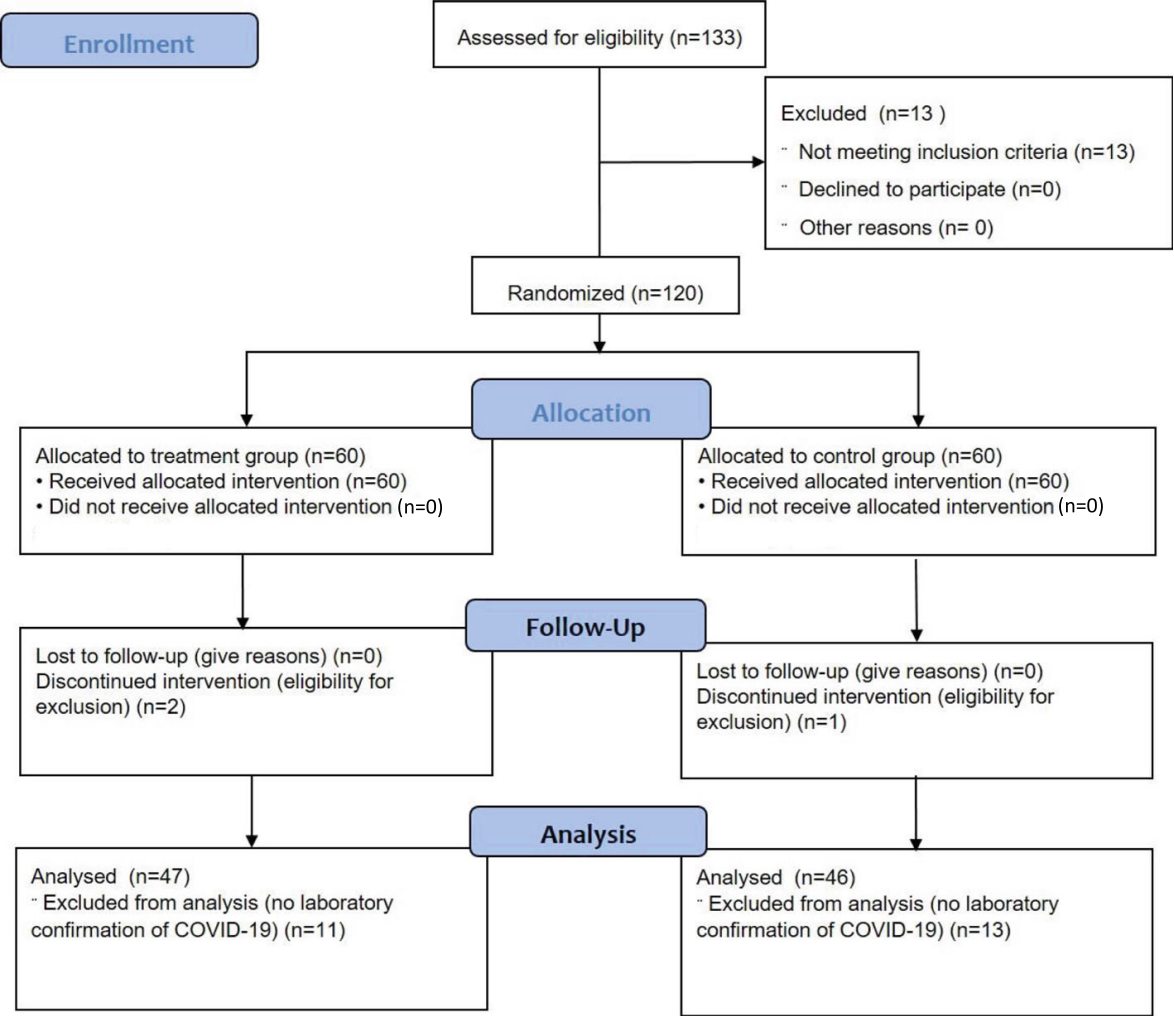
CONSORT diagram: patients included in screening and randomization and excluded from the study

To participate in the study, 133 patients were screened, 120 outpatients aged 18–65 were randomized (Fig. 1).

Thirteen people of the one hundred thirty-three patients screened met the exclusion criteria. The remaining 120 patients with a clinically diagnosed mild coronavirus infection were randomized to either the control group (n = 60) or the main group (n = 60). 3 patients (2.5 %) were excluded from the study. The reason was the presence of exclusion criteria n – 1 from the control group and n – 2 from the main group (loss of contact with the patient). 24 patients (20 %) were excluded from the final assessment of the results (n – 11 in the main group and n – 13 in the control group). The reason was the absence of laboratory verification of COVID-19. Therefore, out of 120 patients randomized from June 2020 to December 2020, 93 patients (77.5 %) (n – 47 in the main group and n – 46 in the control group) with laboratory verification of COVID-19 were considered in the results assessment.

Table 2 shows the gender distribution of the patients in both groups with laboratory verification of COVID-19: 16 men (32.65 %) and 33 women (67.35 %) out of 49 patients in the main group, 15 men (31.91 %) and 32 women (68.09 %) out of 47 patients in the control group.

**Table 2:** Gender distribution of the patients

Gender	Main group (***n*** = 49)		Control group (n = 47)		Chi-square	***p***-value
	n	%	n	%		
Male	16	32.65	15	31.91	0.006	0.9384
Female	33	67.35	32	68.09		

The conclusion has been made at a significance level of 0.05

In general, there were more women than men in the main and control groups (67.72 % vs. 32.28 %) among the patients with a verified diagnosis of COVID-19. The groups are statistically comparable by gender.

Table 3 shows the age distribution of the patients in both groups: the average age of the patients was 33.08 ± 12.34 in the main group and 33.62 ± 11.75 in the control group.

**Table 3:** Age distribution of the patients

Indicator	Group	Statistical values					
		n	Arithmetic mean	Median	Standard deviation	Min	Max
Age	Main	49	33.08	29	12.34	18	63
	Control	47	33.62	31	11.75	18	65
	**t-statistics**	**Number of degrees of freedom**		**t critical dual-sided**		***p***-value	
	0.2175	94		1.9855		0.8282	

The conclusion has been made at a significance level of 0.05

Generally, there were no significant differences between the demographic characteristics of the patients of the main and control groups at baseline (Day 1) (*p* > 0.05).

### Outcomes and estimation

Table 4 presents a comparative characteristic of the dynamics of mild COVID-19 symptoms in the patients of the main and control groups according to the physician’s assessment on a 4-point scale.

**Table 4:** Comparison of the groups by the dynamics of the main symptoms severity according to the physician’s assessment (% of the Mann-Whitney U test)

Indicator	Day	Main group %	Control group %	Mann–Whitney U test	Wilcoxon’s test W	Statistics Z	***p***-value	Differences significance*
Hyperthermia	V1	100	100	1096.00	2224.00	-0.420	0.675	Non-significant
	V2–V1	-41.4	-7.3	765.50	1990.50	-2.979	0.003	Significant
	V3–V1	-98.0	-89.5	1003.00	2228.00	-1.124	0.261	Non-significant
Myalgia	V1	100	100	1003.00	2131.00	-1.127	0.260	Non-significant
	V2–V1	-24.5	6.1	586.00	1811.00	-4.471	0.000	Significant
	V3–V1	-97.1	-67.6	697.00	1922.00	-3.415	0.001	Significant
Nasal congestion	V1	100	100	1455.00	3285.00	-1.854	0.064	Non-significant
	V2–V1	-17.4	27.2	644.50	1869.50	-4.009	0.000	Significant
	V3–V1	-95.4	-65.4	714.00	1939.00	-3.274	0.001	Significant
Nasal discharge	V1	100	100	959.50	2184.50	-1.539	0.124	Non-significant
	V2–V1	-25.5	-29.7	1062.50	2190.50	-0.743	0.457	Non-significant
	V3–V1	-92.7	-85.7	999.00	2127.00	-1.223	0.221	Non-significant
Coughing	V1	100	100	862.00	2087.00	-1.127	0.215	Non-significant
	V2–V1	43.9	28.1	1126.50	2351.50	-0.203	0.839	Non-significant
	V3–V1	-100.0	-51.0	1083.00	2211.00	-2.560	0.005	Significant
Anosmia	V1	100	100	1129.00	2354.00	-0.192	0.848	Non-significant
	V2–V1	170.0	177.2	1121.50	2346.50	-0.226	0.821	Non-significant
	V3–V1	-11.3	74.7	952.00	2177.00	-3.498	0.000	Significant
Rhinolalia	V1	100	100	1147.00	2372.00	-0.034	0.973	Non-significant
	V2–V1	-13.1	23.0	946.00	2171.00	-1.985	0.004	Significant
	V3–V1	-90.2	-65.1	1021.00	2246.00	-0.994	0.320	Non-significant
Sore throat	V1	100	100	1113.00	2241.00	-0.294	0.769	Non-significant
	V2–V1	-51.6	-34.7	973.00	2198.00	-1.457	0.145	Non-significant
	V3–V1	-100.0	-90.9	1049.50	2274.50	-0.780	0.435	Non-significant

*The conclusion has been made at a significance level of 0.05

There were no significant differences in the main clinical manifestations of the disease, such as pyrexia, myalgia, nasal congestion, nasal discharge, coughing, anosmia, rhinolalia, sore throat, between the patients in the main and control groups at baseline (V1) (*p* > 0.05).

Hyperthermia in the main group patients displayed a significantly faster regression on V2: -41.4 % compared to -7.3 % in the control group (*p* < 0.05). On V3, the difference was insignificant.

The myalgia indicator in the main group patients on V2 decreased by 24.5 %, while the control group patients showed more pronounced myalgia: +6.1 %. On V3, the main group patients had myalgia reduced by 97.1 %, and the control group patients — by 67.6 % (*p* < 0.05). A similar dynamic was observed for nasal congestion: on V2, the symptom was reduced by 17.4 % in the main group, while increasing in the control group: +27.2 %. On V3, the main group patients had myalgia reduced by 95.4 %, and the control group patients — by 65.4 % (*p* < 0.05).

Coughing increased in both patient groups on V2: + 43.9 % in the main group and + 28.1 % in the control group (*p* > 0.05). On V3, the coughing disappeared completely in the main group patients, and it decreased by 51.0 % in the control group patients (*p* < 0.05).

The anosmia severity showed a significant increase on V2 in the patients of both groups: + 170.0 % in the main group and + 177.2 % in the control group (*p* > 0.05). Further on, the sense of smell improved and was reduced by 11.3 % in the main group patients compared to the baseline (on V1). The control group patients also showed a tendency to improve their sense of smell, but it remained by 74.7 % more pronounced than that at baseline on V1.

On V2, the rhinolalia severity indicator improved by 13.1 % in the main group, while it increased by 23.0 % in the control group (*p* < 0.05). However, on V3, the dynamics levelled off, and the symptom severity in the patients did not differ significantly: -90.2 % in the main group and − 65.1 % in the control group (*p* > 0.05).

The sore throat regression dynamics during all visits showed a better tendency in the main group patients: -51.6 % vs. -34.7 % on V2 and − 100.0 % vs. -90.9 % on V3. However, the difference was not significant: *p* > 0.05 during all visits. Table 5 presents a comparative characteristic of the dynamics of the symptom’s severity included in the manifestation scale of mild COVID-19 (pursuant to the patient’s self-assessment).

**Table 5:** Comparison of the symptoms dynamics in the observation groups according to the self-assessment (Mann-Whitney U test)

Indicator	Reference point	Mann–Whitney U test	Wilcoxon’s test W	Statistics Z	***p***-value	Differences significance*
Fever	Day 2–Day 1	1085.50	2310.50	-0.696	0.487	Non-significant
	Day 3–Day 1	1001.50	2226.50	-1.276	0.202	Non-significant
	Day 4–Day 1	812.00	2037.00	-2.584	0.010	Significant
	Day 5–Day 1	806.00	2031.00	-2.594	0.009	Significant
	Day 6–Day 1	774.00	1999.00	-2.807	0.005	Significant
	Day 7–Day 1	824.50	2049.50	-2.434	0.015	Significant
	Day 8–Day 1	868.50	2093.50	-2.103	0.035	Significant
	Day 9–Day 1	892.00	2117.00	-1.928	0.054	Non-significant
	Day 10–Day 1	978.50	2203.50	-1.288	0.198	Non-significant
	Day 11–Day 1	994.00	2219.00	-1.172	0.241	Non-significant
	Day 12–Day 1	1067.50	2292.50	-0.625	0.532	Non-significant
	Day 13–Day 1	1095.50	2320.50	-0.417	0.677	Non-significant
Myalgia	Day 2–Day 1	1069.50	2294.50	-0.883	0.377	Non-significant
	Day 3–Day 1	903.00	2128.00	-2.117	0.034	Significant
	Day 4–Day 1	630.50	1855.50	-4.058	0.000	Significant
	Day 5–Day 1	653.50	1878.50	-3.734	0.000	Significant
	Day 6–Day 1	599.00	1824.00	-4.104	0.000	Significant
	Day 7–Day 1	674.00	1899.00	-3.555	0.000	Significant
	Day 8–Day 1	638.00	1863.00	-3.797	0.000	Significant
	Day 9–Day 1	684.00	1909.00	-3.463	0.001	Significant
	Day 10–Day 1	656.50	1881.50	-3.664	0.000	Significant
	Day 11–Day 1	658.50	1883.50	-3.653	0.000	Significant
	Day 12–Day 1	679.00	1904.00	-3.498	0.000	Significant
	Day 13–Day 1	700.00	1925.00	-3.342	0.001	Significant
Nasal congestion	Day 2–Day 1	1130.00	2355.00	-0.227	0.821	Non-significant
	Day 3–Day 1	867.00	2092.00	-2.465	0.014	Significant
	Day 4–Day 1	720.50	1945.50	-3.400	0.001	Significant
	Day 5–Day 1	669.50	1894.50	-3.637	0.000	Significant
	Day 6–Day 1	596.50	1821.50	-4.160	0.000	Significant
	Day 7–Day 1	608.50	1833.50	-4.055	0.000	Significant
	Day 8–Day 1	616.00	1841.00	-3.979	0.000	Significant
	Day 9–Day 1	629.50	1854.50	-3.877	0.000	Significant
	Day 10–Day 1	643.00	1868.00	-3.781	0.000	Significant
	Day 11–Day 1	691.50	1916.50	-3.415	0.001	Significant
	Day 12–Day 1	720.00	1945.00	-3.200	0.001	Significant
	Day 13–Day 1	725.50	1950.50	-3.155	0.002	Significant
Sore throat	Day 2–Day 1	911.00	2136.00	-2.536	0.011	Significant
	Day 3–Day 1	834.00	2059.00	-2.679	0.007	Significant
	Day 4–Day 1	866.00	2091.00	-2.267	0.023	Significant
	Day 5–Day 1	995.50	2220.50	-1.211	0.226	Non-significant
	Day 6–Day 1	970.50	2195.50	-1.397	0.162	Non-significant
	Day 7–Day 1	1026.50	2251.50	-0.956	0.339	Non-significant
	Day 8–Day 1	1034.50	2259.50	-0.893	0372	Non-significant
	Day 9–Day 1	1038.50	2263.50	-0.858	0.391	Non-significant
	Day 10–Day 1	1078.50	2303.50	-0.555	0.579	Non-significant
	Day 11–Day 1	1069.00	2294.00	-0.628	0.530	Non-significant
	Day 12–Day 1	1116.00	2341.00	-0.271	0.787	Non-significant
	Day 13–Day 1	1100.00	2325.00	-0.391	0.696	Non-significant

*The conclusion has been made at a significance level of 0.05

The main clinical symptoms which are most significant for remote assessment of the outpatient’s condition in isolation are self-reported fever and myalgia which reflect a poor general well-being. Following three days of treatment, the patients demonstrated significant differences in the severity of fever, and these differences persisted until Day 8 of treatment. Starting from Day 9, the indicators did not differ significantly. The myalgia indicators differed significantly in the patients of the groups under comparison, starting from the third day until the end of the observation period. The nasal congestion severity dynamics during self-assessment corresponds to the dynamics as evaluated by the physician (Tables 4 and 5). Significant differences were observed between the groups starting from the third day during the entire observation period.

Sore throat is another diagnostically important symptom in patients with COVID-19. From the second to the fourth day of treatment, there were significant differences between the groups. Starting from Day 5, the differences were insignificant. The dynamics of rhinorrhoea, coughing and anosmia according to self-assessment in the patients of the groups under study did not differ significantly.

**Fig. 2:**
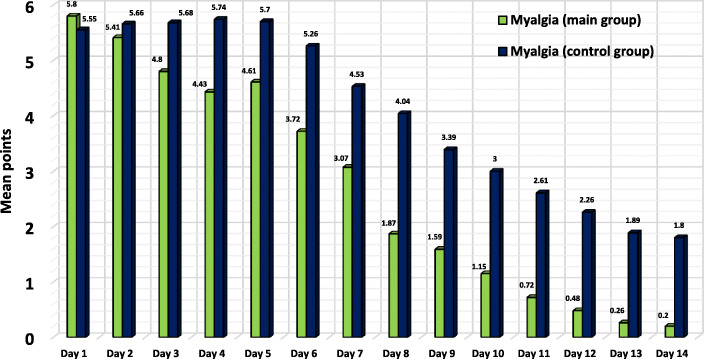
Myalgia severity dynamics in the groups according to the self-assessment results

As is known, the presence of symptoms such as fever and myalgia are an important criterion for assessing the disease severity and one of the main indications for taking non-steroidal anti-inflammatory drugs or antipyretics by the patients. We analysed the dynamics of NSAID or antipyretics intake subject to the severity of fever and/or myalgia based on the results of the patients’ self-assessment (Fig. 2).

**Fig. 3:**
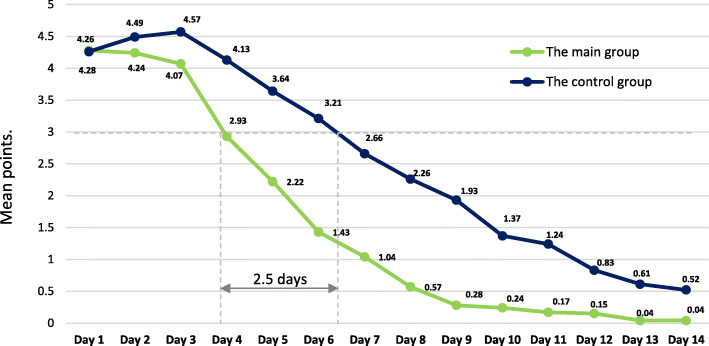
Fever severity dynamics in the groups according to the self-assessment results

As can be seen from the Fig. 3, fever of moderate severity (more than 3 points), requiring the prescription of antipyretics, was observed in the patients of both groups during the first three days of the disease. Starting from Day 4, the main group patients reported a decrease in fever less than 3 points, which made NSAID intake superfluous. The control group patients featured similar indicator values starting from Day 6.5. Besides, the control group displayed a tendency towards increased hyperthermia in the first three days of observation.

The myalgia severity of more than 3 points, requiring the acetaminophen or NSAID intake, lasted up to Day 7 in the main group patients and up to Day 10 in the control group patients. Furthermore, the control group demonstrated a tendency towards increasing the myalgia severity from 5.55 points on the first day to 5.74 points on the fourth day. Therefore, the therapeutic benefit in the treatment of hyperthermia and myalgia symptoms because of BNO 1030 intake is three days.

The patient self-isolation regime provided for the possibility of remote consulting. The patients had the possibility of telephone contact with a physician at any time, therefore, the number of additional telephone contacts can serve as a criterion for the effectiveness of treatment. The number of additional consultative contacts with a physician in the control group is significantly higher than that in the main group: 32 vs. 16. The most common causes of remote consulting were hyperthermia and myalgia.

We have compared the treatment outcomes (e.g. needed hospitalization, further duration of sickness etc.) between the groups on the 14th day, i.e. by the end of the self-isolation period (Table 6).

**Table 6:** Group analysis by treatment outcome

Result	Main group (n=47)		Control group (n = 46)		Chi-square	***p***-value
	n	%	n	%		
**Convalescence**	45	96.6	34	73.9	-	0.0418*
**Hospitalization**	2	4.4	4	8.6		
**Keeps on being sick**	-	-	8	17.3		

The conclusion has been made at a significance level of 0.05

*Comparison performed using Fisher’s exact test

As of the fourteenth day, 45 (96.6 %) out of 47 main group patients recovered, 2 patients (4.4 %) were hospitalized. 34 (73.9 %) out of 46 control group patients recovered, and 4 patients (8.6 %) were hospitalized. Moreover, 8 patients (17.3 %) in the control group complained about the persisting of residual symptoms (from 1 to 3 points according to 10-point VAS) at Day 14 (end of treatment). Indicators for the treatment outcome criterion were significantly different (*p* < 0.05).

### Safety and tolerability

The analysis of the safety and tolerability shows that the therapy was well tolerated. During the third, final visit, two patients from the treatment group and one patient from the control group noted that they experienced temporary symptoms of abdominal discomfort: nausea, heartburn, moderate pain. One patient in the treatment group reported an episode of diarrhoea. All these symptoms were mild and did not lead to a change in the order of taking medications.

## Discussion

The present study has demonstrated that women prevailed over men (67.72 % vs. 32.28 %) among the patients with mild COVID-19, and the average age of the patients was 33.35 y.o. ± 12.04. COVID-19 in males is associated with a greater severity of the disease and higher mortality rates, according to data published [8, 32]. The proportion of men with a severe course of the disease in different age groups was also higher than that of women [32–34]. It has also been shown that age also influences the outcomes of patients hospitalized with COVID-19: the elderly is more likely to have a severe disease form. Therefore, there are important age-gender differences between groups of patients with different severity of the disease, and such patients require different approaches to care in terms of possible outpatient treatment in the patient’s self-isolation mode.

Recommendations for the pharmacotherapy for mild COVID-19 include only symptomatic agents to relieve symptoms like, antipyretics (acetaminophen or ibuprofen) [1, 2]. At the same time, in the case of non-severe acute viral infections of the upper respiratory tract, some phytopreparations have shown proven efficacy [30, 31]. In this regard, there is a need to carry out studies on the effectiveness of phytotherapeutic agents valid in terms of GCP compliance, in particular BNO 1030, in the treatment of a mild form of coronavirus disease.

The present study has demonstrated that the use of the phytopreparation BNO 1030 in addition to the standard symptomatic therapy can be effective. Patients in the BNO 1030 group demonstrated a significant decrease in the severity of symptoms such as hyperthermia, myalgia, nasal congestion, coughing, anosmia, rhinolalia, assessed by a physician on a 4-point scale on V2 and V3 in comparison with V1 and the control group. The symptom regression dynamics and the results of the patient’s self-assessment have shown significant differences.

An important and interesting conclusion of the study is that the control group patients, in contrast to the main group patients, featured an increase in the severity of clinical symptoms, in particular hyperthermia and myalgia, in the first 3–4 days of observation. This led to more frequent additional remote consultation of the patients. Many researchers are of the opinion that the weak regression dynamics, and, even more so, the increase in the intensity of acute respiratory infection symptoms, is the driving force of the unjustified prescription of antibiotics among doctors and the desire for antibiotic therapy among patients themselves, which is one of the main reasons for the global issue of antibiotic resistance [35]. Pursuant to several studies, more than 90 % of patients with COVID-19 receive antibiotics, including combination therapy and parenteral drugs on an outpatient basis. In this regard, WHO recommends antibacterial therapy only in a hospital setting in the case of bacterial infection [36].

The results obtained by us in the treatment of mild COVID-19 using BNO 1030 are consistent with the data published in the literature [30, 31]. The results of these studies indicate that BNO 1030 (Imupret®) is effective for the treatment of acute respiratory viral infections. Our results are supported by data from a German observational study which demonstrated the efficacy and safety of the drug in more than 1100 patients with recurrent acute upper respiratory tract infections [37].

An important and interesting finding of this study is that due to the more pronounced regression of symptoms such as fever and myalgia, patients in the BNO 1030 group needed fewer systemic NSAIDs (ibuprofen) or acetaminophen. The therapeutic benefit is three days. Several researchers have expressed concern that ibuprofen might lead to a more severe course of coronavirus disease [3, 4]. Reduced duration of NSAID intake, together with other effects of the medicinal product, is the key to success in improving the treatment results. The main group patients displayed significantly better values for the treatment outcome criterion. As of the fourteenth day, 96.6 % of the main group patients recovered, and 4.4 % were hospitalized. In the control group, 73.9 % of the patients recovered, and 8.6 % were hospitalized. Furthermore, 17.3 % of the control group patients still preserved the symptoms on the 14th day (end of treatment) and required further pharmacotherapy and observation.

Therefore, the BNO 1030 efficacy shown in this study is broadly consistent with the results of earlier studies in patients with acute viral infections. However, its advantage is the diagnosis of mild COVID-19 established according to the accepted criteria. The group of patients randomized and included in the analysis, homogeneous in diagnosis and clinical manifestations, allowed to draw reasonable conclusions on the assessment of the overall treatment results. The number of patients recovered is significantly higher in the study group than in the control group. The “therapeutic gain” according to the criterion of hyperthermia and myalgia symptomatic treatment in the main group patients is 3 days, which reflects a significant advantage in the number of patients recovered. This allows for reducing the number of patients with continued illness and the need for further isolation and pharmacotherapy until complete regression of symptoms. This is critical in terms of infection spread since reducing the average duration of the infection period can prevent an average of 442,852 to 44.4 million cases of SARS-CoV-2, varying the proportion of cases treated, the average duration of the infectious period, and the virus reproductive capacity. Providing treatment for up to 75 % of all infected cases, including asymptomatic infections with R_0_ of 2.5, is assumed to prevent 35.9 million cases and 4 million hospital stays, saving USD 48.8 billion [38].

The design provided for a comparative study without placebo control. The comparison was carried out with treatment according to clinical guidelines providing for the mandatory prescription of symptomatic treatment alone, using antipyretics [1, 2]. In this regard, all the differences in treatment results can be attributed to the clinical effects of BNO 1030.

### Limitations

Limitations of the study include open label study design due to unavailability of placebo. The diagnosis was initially made based on clinical data and, only after a few days, laboratory data became available. The limited number of patients in the study did not allow assessing the effect of additional intake of the herbal preparation on the risk of developing severe forms of the disease.

## Conclusions

The additional intake of some herbal drugs such as BNO 1030 (Imupret®) can be beneficial for the treatment of mild COVID-19, significantly relieving clinical symptoms, improving the assessment of the patient’s general condition, reducing the treatment duration and the need for antipyretics with an acceptable safety profile. The medicinal product can be recommended to be included in the treatment regimen for patients with mild COVID-19 however, to better assess the effect of such treatment on the symptoms of the disease individually, as well as on issues related to safety, additional studies with a large number of patients and placebo control are required.

Further research will aim at investigating the medicinal product effect on the virus isolation period.
